# Reproducibility of fetal ultrasound doppler parameters used for growth assessment

**DOI:** 10.1007/s00404-024-07883-7

**Published:** 2025-01-16

**Authors:** Raghda Zidan Sweid, Vera Donadono, Davide Casagrandi, Laura Sarno, George Attilakos, Pran Pandya, Raffaele Napolitano

**Affiliations:** 1 Fetal Medicine Unit, Elizabeth Garrett Anderson Wing, University College London Hospitals NHS Foundation Trust, 250 Euston Road, London, NW1 2BU UK; 2 Institute for Women’s Health, Elizabeth Garrett Anderson, University College London, London, UK; 3 Department of Neurosciences, Reproductive Science and Dentistry, University of Naples “Federico II”, Naples, Italy

**Keywords:** Pulsatility indices, Umbilical artery, Middle cerebral artery, Cerebro-placental ratio and umbilical cerebral ratio

## Abstract

**Objectives:**

To produce standards of references for quality control and assess the reproducibility of fetal ultrasound Doppler measurements commonly used for blood flow assessment in fetal growth.

**Methods:**

Women with singleton normal pregnancies were prospectively recruited at University College London Hospital, UK, between 24 and 41 weeks. Umbilical artery (UA), middle cerebral artery (MCA), and their pulsatility indices (PI), resistance indices (RI) and ratios such as cerebro-placental (CPR) and umbilical cerebral ratio (UCR) were obtained twice by two sonographers in training or after completion of training, blind to each other’s measurements. Bland–Altman plots were generated, the mean differences and 95% limits of agreement (LOA) were calculated to assess intra- and interobserver reproducibility. Values were expressed as absolute values or as z-score.

**Results:**

One hundred ten women were recruited. Overall reproducibility was variable for absolute values and highly variable for z-scores, independently from vessel sampled, index or ratio used, intra- or interobserver reproducibility. The widest absolute values of 95% LOA were 0.3 for UA PI, 0.7 for MCA PI, 0.9 for CPR and 0.3 for UCR, respectively. Regarding z-score, the widest 95% LOA were 1.9 for UA PI, 2.1 for CPR and 1 for UCR. Reproducibility was slightly better for intra- compared with interobserver variability. There was significant difference in z-score reproducibility between MCA peak systolic velocity and CPR vs UCR.

**Conclusions:**

Reference standards of reproducibility of fetal Doppler parameters are produced for standardization and quality-control purposes. Overall, the reproducibility for fetal Doppler parameters was variable independently from vessel sampled, Doppler index (PI or RI) or ratio used, intra- and interobserver comparison. UCR was the most reproducible parameter which should be recommended, together with UA PI, for clinical use and in research studies on fetal growth.

**Supplementary Information:**

The online version contains supplementary material available at 10.1007/s00404-024-07883-7.

## What does this study add to the clinical work

**Table:** 

We produced reference standards of reproducibility for fetal Doppler parameters for quality control purposes. Umbilical artery pulsatility index and umbilical cerebral ratio are highly reproducible parameters and they should be recommended for clinical use.	

## Introduction

Doppler assessment of the fetal umbilical and cerebral circulation is essential in the diagnosis and management of fetal growth restriction (FGR) and anemia [1–4]. Cerebral blood-flow redistribution (otherwise known as ‘brain sparing’) indicates preferential fetal cardiac output toward the brain, heart and adrenal glands in the presence of hypoxemia [5]. It can be evaluated through the assessment of fetal middle cerebral artery (MCA) pulsatility index (PI), resistance index (RI), or the ratio between MCA PI and umbilical artery (UA) PI, namely the cerebroplacental ratio (CPR) and umbilical cerebral ratio (UCR) [6].

Despite national and international guidelines recommend the use of fetal Doppler, there is no international consensus on what chart to use and what strategies to adopt to reduce the variability of measurements [7–9]. The International Society of Ultrasound in Obstetrics and Gynecology (ISUOG) recommends implementing a quality-control system to reduce measurements variability, however it does not recommend a specific standard for reproducibility assessment [10].

Variability of measurements does have an impact on clinical care and contributes to the heterogeneity of centiles ranges reported in studies producing fetal charts [11].

Reproducibility of Doppler measurements has been previously reported either in studies aimed at creating charts or in small series with limited information on maternal and fetal characteristics of the study population. A high risk of bias in the methodology used was reported [12, 13].

The aim of this study is to report the intra- and interobserver reproducibility of fetal Doppler parameters, including UA PI and RI, MCA PI, RI, and PSV, and their ratios CPR and UCR, in a prospective cohort of normal singleton pregnancies throughout gestation and to produce reference standards for quality-control purposes.

## Materials

Pregnant women with singleton non-anomalous fetuses were included in this study as part of a hospital service improvement project. Fetuses with estimated weight based on the Hadlock’s formula less than the 10th centile or with abdominal circumference centiles drop of more than 50 centiles since 20 weeks were excluded. Scans were performed between 24 and 41 weeks ensuring an equal distribution across all weeks of gestation at the same time of attending for routine or clinically indicated scan appointments in the Ultrasound Screening Unit at University College London Hospital, London, UK, between March 2021 and March 2022.

The gestational age was calculated based on crown-rump length obtained at 11–14 weeks of gestation or according with embryo transfer details in cases of in-vitro fertilization. All ultrasound scans were performed by sonographers in training who acquired competences in performing growth scans after 4–6 months of training (junior) or with either more than 3000 scans or more than 1 year of experience (senior), according with a dedicated programme and international standards of training [14, 15]. Eight different machines from the same manufacturer were used in the study (Voluson E8, GE, Austria) using the transabdominal probe.

Each woman was examined by two sonographers (one junior and one senior).

Fetal UA, MCA Doppler PI and RI, and their ratios (CPR: MCA PI/UA PI and UCR: UA PI/MCA PI) were obtained prospectively by two sonographers blindly to each other measurements. Doppler measurements were obtained twice by each operator. As soon as the second set of measurements was obtained by the first operator, the second operator entered the room and acquired its measurements in the same sequence. Each woman was included only once in the study.

To obtain the measurements, standards of quality control were followed. Briefly: for UA, a free loop of umbilical cord was obtained and magnified to fill 50% of the screen with zoom box and sample gate in center of vessel. Four to six waveforms with consistent and similar signal were recorded with 75% of peak systolic velocity (PSV) fitting the scale range [16, 17].

For MCA, an axial section of the brain, including the thalami and the sphenoid bone wings, was obtained and magnified, then color flow mapping was used to identify the circle of Willis and the proximal MCA and pulsed-wave Doppler gate was placed at the proximal third of the MCA, close to the origin of the internal carotid artery. The angle between the ultrasound beam and the direction of blood flow was kept as close as possible to 0 degrees and adjusted for after acquisition. At least three clear consecutive waveforms were recorded [18]. PSV and indices were calculated using either manual or auto-trace facility. Bland–Altman plots were created, and analyses were conducted utilizing Microsoft Excel^®^ (Microsoft^®^ Corporation 2007, Washington, DC, USA) and statistical package SPSS^®^ Statistics version 25.0 (IBM Corp., Armonk, NY, USA). For the intraobserver reproducibility the first and second measurements of each operator were compared. Interobserver reproducibility was determined by comparing the first measurements of one operator with those of the other operator, and the same procedure was repeated for the second measurements. Mean difference and 95% limits of agreement (LOA) were computed for all comparisons. Values were expressed as absolute values and as z-score, when relevant references developed in studies at low risk of bias were available such as for UA PI [19] and UA RI [19], MCA PI [20] and PSV [20], CPR [21] and UCR [22]. Outliers with ≥ 2.5 standard deviations from the mean difference were excluded. Means of the 95% LOA duplicate measurements were compared and a difference was considered significant if p value was < 0.05 using paired sample t-test, between intra- vs interobserver reproducibility, UA PI vs UA RI, MCA PI vs MCA RI, and CPR vs UCR. z-scores were utilized when available otherwise absolute values were used for t-test comparison when z-scores were not available (as for MCA RI) [23].

A power calculation was performed to obtain adequate sample size according with previous reports and we estimated that a total of 90 measurements would be needed to detect a 20% difference in 95% LOA z-score in any of the planned analysis with 90% power (alpha 0.05). A sample of more than 100 women was considered adequate to achieve this [24, 25].

The study was conducted as a planned audit for the purpose of monitoring the training performance. Individual patient consent was therefore not required. Data were anonymized. We have interrogated the Health Research Authority tool-UK and the current study was considered not to require ethical approval.

## Results

Between March 2021 and March 2022, a total of 110 women with a singleton pregnancy, between 24 and 41 weeks, were included. Doppler measurements of UA and MCA were obtained in all cases for a total of 880 acquisitions (3080 values analyzed). Maternal demographics of the population recruited are reported in Table 1 confirming a low-risk population. Overall, the 95% LOA reproducibility for fetal Doppler parameters were variable with absolute values of PI, RI or ratios ranging between 0.1 and 0.9. This resulted in a higher variability regarding z-score (0.8–2.1), independently from the vessel sampled, index or ratio used and intra- vs interobserver comparison (Table 2, Supplementary Fig. 1). There was no difference between the intraobserver reproducibility of measurements obtained by junior or senior sonographer, so data are reported as a single group analysis for intraobserver reproducibility.

**Table 1:** Maternal demographics

Characteristics	Population = 110
Maternal age, years	34/4.8 (23–48)
Body mass index, Kg/m^2^	23.4/4.3 (17–37)
Parity	1/1.3 (0–8)

Numbers are expressed as median/standard deviation (range)

**Table 2:** Intra- and inter-observer reproducibility of fetal Doppler

		Mean difference	95% Limits of agreements
		Absolute value/z-score	Absolute value/z-score
UA			
PI	Intraobserver	0.02/0.13	0.25/1.65
	Interobserver	0.03/0.19	0.30/1.94
RI	Intraobserver	0.01/0.08	0.10/1.53
	Interobserver	0.01/0.16	0.13/2
MCA			
PI	Intraobserver	0/− 0.01	0.55/1.31
	Interobserver	0/− 0.01	0.73/1.77
RI	Intraobserver	0	0.10
	Interobserver	0	0.14
PSV	Intraobserver	0.85/0.09	11.79/1.3
	Interobserver	− 0.03/− 0.02	18.37/2
CPR	Intraobserver	− 0.04/− 0.18	0.73/1.67
	Interobserver	− 0.06/− 0.14	0.95/2.13
UCR	Intraobserver	0.01/0.05	0.21/0.79
	Interobserver	0.02/0.09	0.26/0.97

*UA* umbilical artery, *PI* pulsatility index, *RI* resistance index, *MCA* middle cerebral artery, *PSV* peak systolic velocity, *CPR* cerebro-placental ratio, *UCR* umbilical cerebral ratio

The 95% LOA absolute values of PI were narrower for UA for both intra- and interobserver reproducibility (0.2 and 0.3, respectively) compared to MCA results (0.5 and 0.7, respectively), while calculating z-scores the range of reproducibility was wider with less difference between the two vessels (1.6 and 1.9 for UA versus 1.3 and 1.8 for MCA, intra- and interobserver reproducibility, respectively).

The 95% LOA of the PSV in absolute values were relatively narrow for MCA for both intra- and interobserver reproducibility (11.8 and 18.3, respectively); however, z-scores resulted in a wider range (intra- and interobserver LOA were 1.3 and 2, respectively).

As expected, measurements showed a better result for intra- than for interobserver reproducibility, reaching significance only for MCA PSV. The 95% LOA of the UA and MCA PI ranges were 0.2 and 0.5 (z-score: 1.3–1.6) and 0.3 and 0.7 (z-score: 1.7–2) for intra- and interobserver reproducibility, respectively.

**Fig. 1:**
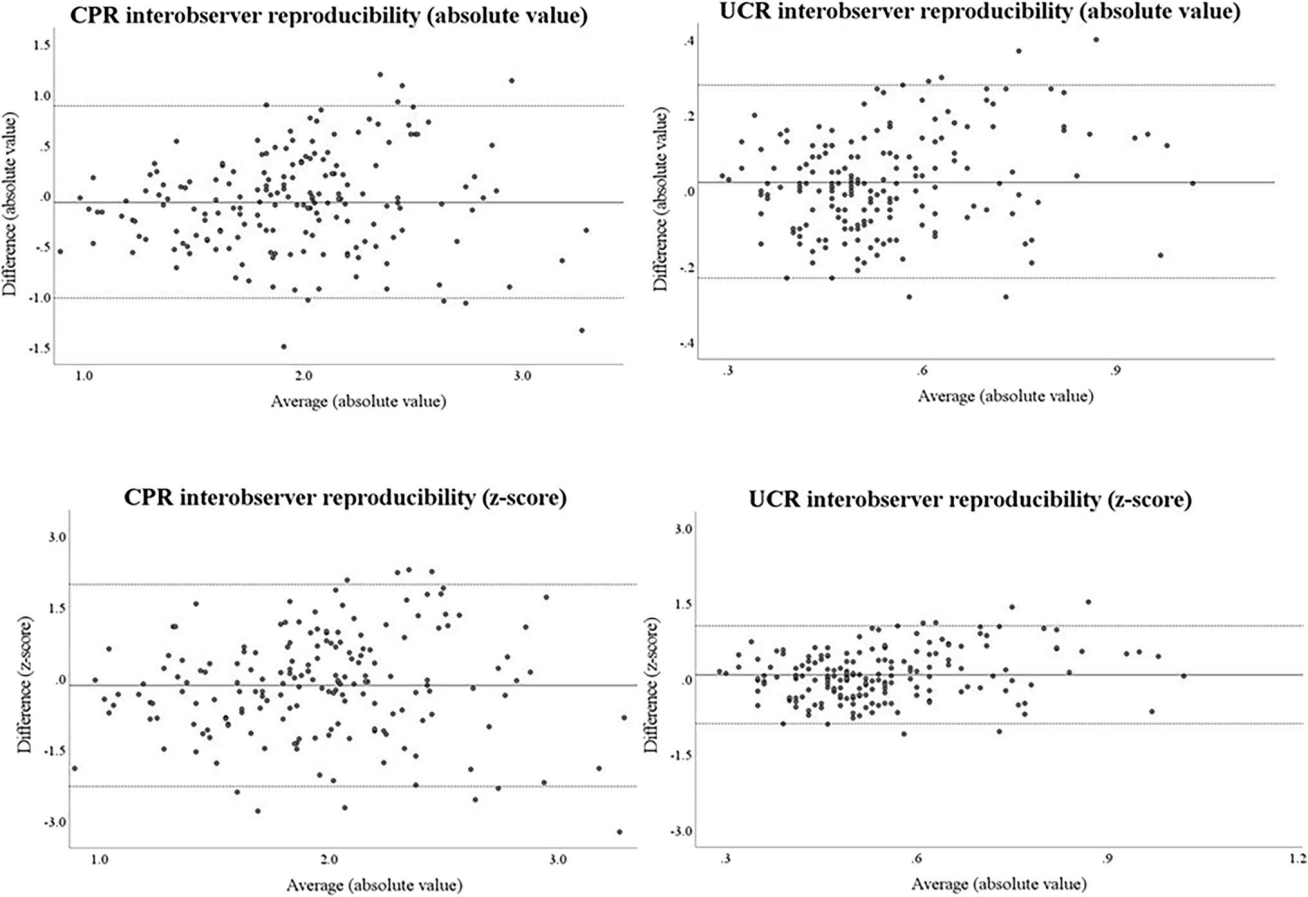
Interobserver reproducibility for fetal cererbo-placental (CPR) and umbilical cerebral (UCR) Doppler ratio expressed in absolute values and z-scores

The above applied also to Doppler ratios, with UCR having better reproducibility than CPR. The 95% LOA were 0.7 (z-score: 1.7) for CPR versus 0.2 (z-score: 0.8) for UCR, and 0.9 (z-score: 2.1) for CPR versus 0.3 for UCR (z-score: 1), for intra- and interobserver reproducibility, respectively (Fig. 1, Table 2). UCR z-score was also narrower than MCA PI z-score. The t-test showed a statistically significant result in the mean differences when comparing the intra- vs the interobserver reproducibility of the MCA PSV (*p* value = 0.034), and when comparing the intraobserver reproducibility of the CPR vs the UCR (*p* value = 0.042) (Table 3).

**Table 3:** Paired sample t-test on all comparisons of means of the 95% limits of agreement duplicate measurements

		*p* value		*p* value
UA	PI intra- vs interobserver	0.147	PI vs RI intraobserver	0.175
	RI intra- vs interobserver	0.128	PI vs RI interobserver	0.104
MCA	PI intra- vs interobserver	0.56	PI vs RI intraobserver	0.176*
	RI intra- vs interobserver	0.42*	PI vs RI interobserver	0.998*
	PSV intra- vs interobserver	0.034		
CPR	intra- vs interobserver	0.599	CPR vs UCR intraobserver	0.042
UCR	intra- vs interobserver	0.586	CPR vs UCR interobserver	0.115

*UA* umbilical artery, *PI* pulsatility index, *RI* resistance index, *MCA* middle cerebral artery, *PSV* peak systolic velocity, *CPR* cerebro-placental ratio, *UCR* umbilical cerebral ratio

^*^T-test on absolute values, otherwise *t*-test performed on z-score

## Discussion

The aim of our study was to assess the reproducibility of fetal Doppler parameters commonly used for fetal growth assessment to produce standards of reference for quantitative quality control.

The data from our study showed that the results of reproducibility assessment for fetal Doppler parameters were variable, independently from vessel sampled, index or ratio used, intra- or interobserver assessment.

Despite our near optimal study conditions, measurements are highly variable, probably due to fast changes in the hemodynamics of the fetus, leading to different results even few minutes apart [26, 27].

Previous studies reported on reproducibility of fetal Doppler parameters, however not all parameters were analyzed in the same low-risk population, there was not a consistent approach in acquiring measurements and quantitative analysis was not reported using Bland–Altman plots producing mean and 95% LOA [17, 20, 26–35]. Most studies report on intraclass correlation coefficient, reliability index or coefficient of variation [23–26, 26–28, 28–33]. The former is a measurement of agreement rather than reproducibility. The latter two can estimate variation of measurements, but they do not provide quantitative values which can be used in clinical practice. Surprisingly not all studies report LOA. Providing mean differences can explain what the reproducibility would be on average if multiple measurements were obtained (> 100). However, in clinical practice a measurement is repeated only once or twice, by the same or a different operator. For this reason, it is important to report the 95% LOA which provides an estimate of what the variability would be if measurement is repeated only once (random error, rather than the systematic error).

UA PI has been reported to be highly variable, especially at different sample sites of the cord [27]. In studies where Bland–Altman plots were produced (Table 4), UA PI 95% LOA varies from 0.2 to 0.3 [27, 30]. In our cohort we reported similar PI values (0.2 and 0.3 for intra- and interobserver reproducibility, respectively).

**Table 4:** Quantitative reproducibility of fetal Doppler values and z-scores in previous studies and current study

	Intraobserver reproducibility				Interobserver reproducibility			
	Absolute values		z-score		Absolute values		z-score	
	Mean difference	95% LOA	Mean difference	95% LOA	Mean difference	95% LOA	Mean difference	95% LOA
UA PI	0.02^††^ 0.01^†^	0.25^††^ 0.24^†^	0.13^††^	1.65^††^	0.03^††^	0.3^††^ 0.34^¶^	0.19^††^	1.94^††^
MCA PI	0.0^††^ 0.00^**^ 0.01^†^	0.55^††^ 0.24^**^ 0.58^§^ 0.38^†^	0.01^††^	1.31^††^ 2.3^§^	0.01^††^ 0.05^**^ 0^*^	0.73^††^ 0.84^**^ 0.51^*^ 0.58^§^	0.01^††^	1.77^††^ 1.8^§^
CPR	0.04^††^ 0.12^‡^	0.73^††^ 1.34^‡^	0.18^††^	1.67^††^	0.06^††^	0.95^††^	0.14^††^	2.13^††^
UCR	0.01^††^	0.21^††^	0.05^††^	0.79^††^	0.02^††^	0.26^††^	0.09^††^	0.97^††^

Values are expressed in mean difference and 95% Limits of agreements (LOA). If a positive and a negative 95% LOA is provided, the mean of the absolute value of the two is calculated

*UA* umbilical artery, *PI* pulsatility index, *RI* resistance index, *MCA* middle cerebral artery, *PSV* peak systolic velocity, *CPR* cerebro-placental ratio, *UCR* umbilical cerebral ratio

Modified from Zidan 2024^††^, Figueras 2006^¶^, Segev 2021^†^, Ebbing 2007^**^, Salvi 2015^*^, Pasquini 2020^§^ (z-score derived from centiles), Bhide 2019.^‡^ (absolute values derived from Bland–Altman plots)

Conversely, no studies report on z-score reproducibility which is important as it provides an estimation of the implication on centiles calculation. In our cohort narrow absolute PI 95% LOA corresponded to wide z-scores (1.6–1.9). Small differences in absolute values can translate into significant differences in z-score and centiles calculated, and this depends on what chart is used.

We found similar findings for MCA indices, CPR and UCR ratios. 95% LOA of MCA PI absolute values vary from 0.2 to 0.8 [20, 29, 30, 36]. Similarly to UA, we observed good reproducibility for absolute PI values (0.5 and 0.7 for intra- and interobserver reproducibility, respectively), however wide range of z-scores (1.3–1.7).

Absolute 95% LOA of CPR can reach 1.34 [37]. In our cohort we reported narrower PI values (0.7 and 0.9 for intra- and interobserver reproducibility, respectively), which again translates into highly variable z-scores (1.7–2.1). UCR was more reproducible than CPR (0.2 and 0.3 for intra- and interobserver reproducibility, respectively, z-score: (0.8 -1).

Many 95% LOA were closer to 1.6 z-score which implies that one value could plot normally or abnormally on a chart (< 5th or > 95th centile), only due to variability in measurements obtained, rather than due to true biologic differences. As the z-score values depend on the chart used, the higher the risk of bias in the methodology used in studies creating fetal charts, the more variable is the range of centile produced [11, 38]. Effort should be put in place to standardize practice in using a single chart generated from studies with high methodological quality [39].

There was no significant difference between index used for UA and MCA (PI and RI), so we would support the use of PI, as recently published prescriptive charts are available [19] and PI is validated in studies for the management of FGR [40–44].

In the TRUFFLE study it is reported that UCR compared with CPR can better discriminate fetuses with an abnormal outcome. We provide another reason to choose UCR as it appears to be more reproducible [6]. This is difficult to explain, however the fact that using UCR there is a trend to infinite mathematical ratio could be related to the findings. CPR otherwise tends to 0 at extreme abnormal values. Therefore, the range of values of CPR is smaller than UCR. As a consequence, highly variable measurements have a higher impact on CPR centiles calculation than for UCR. Furthermore, in the Truffle 2 feasibility study, UCR absolute values perform similarly, if not better, to CPR centiles in discriminating growth restricted babies with abnormal outcome [2]. In facts, gestational age specific absolute thresholds, rather than centiles, are used as criteria for delivery in the TRUFFLE 2 randomized trial (UCR > 1.0 at 32w + 0d—33w + 6d and > 0.8 at > 34w + 0d) [41].

The use of UA, MCA or their ratio is recommended for the management of FGR [7–9]. International guidelines recommend to perform a quality control on fetal Doppler measurements [10, 17], without recommending specific standards. The high methodological quality of the current study can provide a standard of reference for monitoring performance.

As expected, data from this study confirm that intra- is better than interobserver reproducibility [12, 14, 23, 24].

There are few limitations in this study. First, the relatively small sample studied, however, the statistical power was adequate. It is possible that a larger sample size might either reduce or indeed increase the variability observed.

Second, it could be argued that acquiring the same measurement multiple times during the same scan might increase the reproducibility between initial and last acquisition. However, often the measurements have not been obtained consecutively, due to the fetus changing position or to allow the operator to acquire images needed for routine care. Finally, multiple operators participated in the study. This can be seen as a potential bias, nevertheless, we consider this more representative of real clinical practice scenarios.

Our reproducibility values are better than reported before.

This could be due to setting up a study with near optimal conditions: selection of low-risk women, the acquisition of measurements according to a standardized protocol and the experience of the sonographers. The above could be seen as a limitation creating an artificial scenario, however this is set up to minimize the impact of other variables on reproducibility to produce reference standards.

In conclusion, reference standards of reproducibility for fetal Doppler parameters are produced that can be used for quantitative quality control and assessment of sonographer performance. Fetal Doppler indices have significant variability, especially when calculating z-score. The above is independent from vessel sampled, index or ratio used and intra- or interobserver reproducibility. We propose to use UA PI and UCR as prescriptive charts are available and the above parameters are used in randomized trials [19, 42–44]. It is important to introduce quality-control programmes in such studies due to the high impact of reproducibility on clinical thresholds for abnormality. For example, in the RATIO37 trial between 5 and 6% of the population had a CPR < 5th centile. We observed that the intraobserver reproducibility was 1.67 z-score (around 95th and 5th centile) for CPR and 0.79 (around the 73rd and 23rd centile) for UCR, respectively. The implications for clinical practice are as such that the same operator can acquire a repeated measurement 45 centiles (95 or 5 centiles – 50 mean centile) or 23 or 27 centiles (73 or 23 centiles—50 mean centile) above or below the average reproducibility value for CPR and UCR, respectively [45]. Variation due to reproducibility can imply that more than 50% (5 + 45%) or less than 1% (5–50%) of fetuses would meet criteria for a CPR < 5th centile in the RATIO37 population. If we assume a similar rate of fetuses meeting threshold for abnormality for a UCR > 95th centile (5%), Less than 1% (5 – 23%) or more than 32% (5 + 27%) of fetuses would meet criteria for abnormality due to reproducibility impact.

Thresholds for abnormality are similar using either the UCR 95th centile from Arduini et al. [22] or the CPR 5th centile from Baschat et al. [21, 44], therefore they can be both be used in clinical practice.

## Conclusion

We produced reference standards of reproducibility for fetal Doppler parameters for quality-control purposes. Overall, the reproducibility for fetal Doppler parameters was variable independently from vessel sampled, Doppler index (PI or RI) or ratio used, intra- and interobserver comparison. UCR was the most reproducible parameter which should be recommended, together with UA PI, for clinical use and in research studies on fetal growth.

## Supplementary Information

Below is the link to the electronic supplementary material.

Supplementary file1 (DOCX 1245 KB)

## Data Availability

The data that support the findings of this study are available on request from the corresponding author. The data are not publicly available due to privacy or ethical restrictions.
