# Supplementary material for: Reproducibility of fetal ultrasound doppler parameters used for growth assessment
Source: Arch Gynecol Obstet. 2025 Jan 16;311(3):669–76. doi: 10.1007/s00404-024-07883-7 (PMC11919989; doi:10.1007/s00404-024-07883-7)

**Supplementary Figure 1:** Bland-Altman plots for intra- and interobserver reproducibility of fetal Doppler Pulsatility (PI) and Resistance Indices (RI), Peak Systolic Velocity (PSV), reported in absolute values and z-scores for: A) Umbilical Artery (UA), B) Middle Cerebral Artery (MCA), C) Cerebro-Placental Ratio (CPR), and D) Umbilical-Cerebral Ratio (UCR)

A) UA


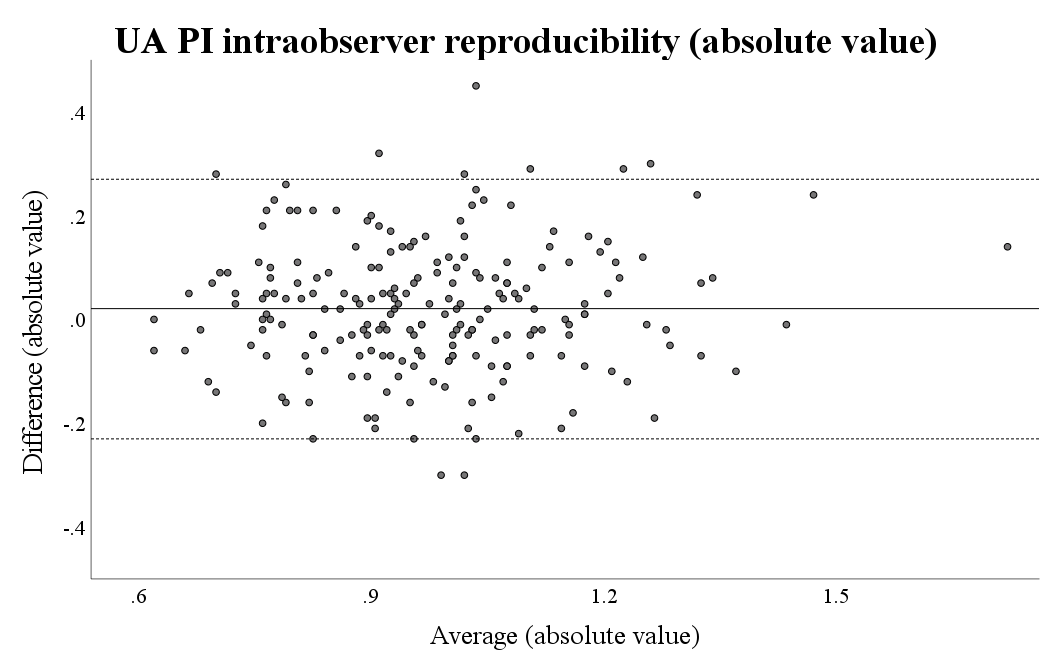

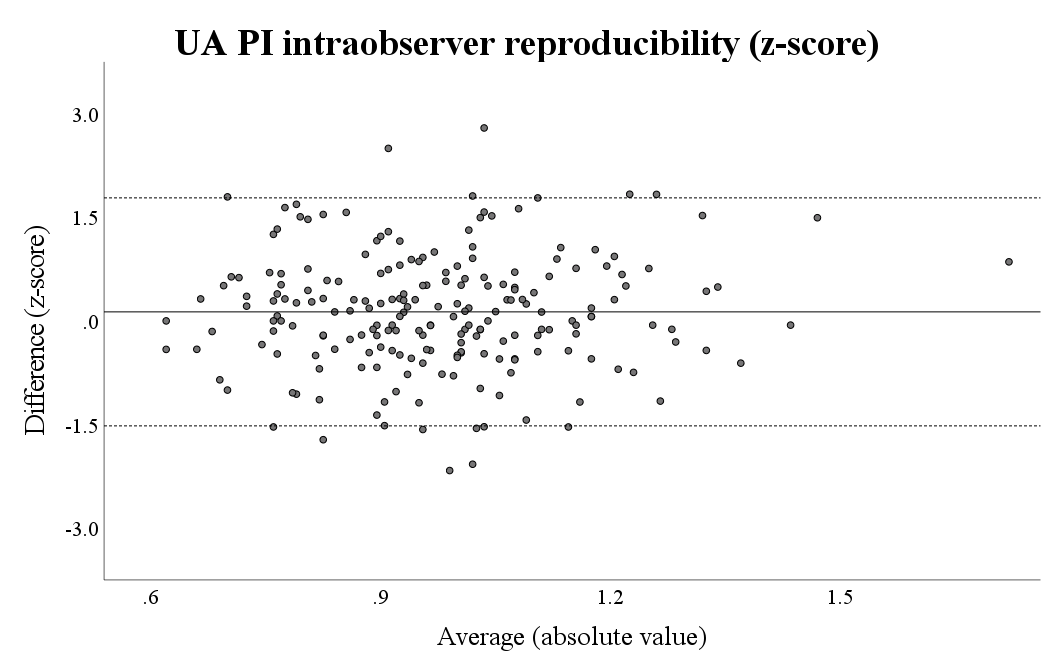

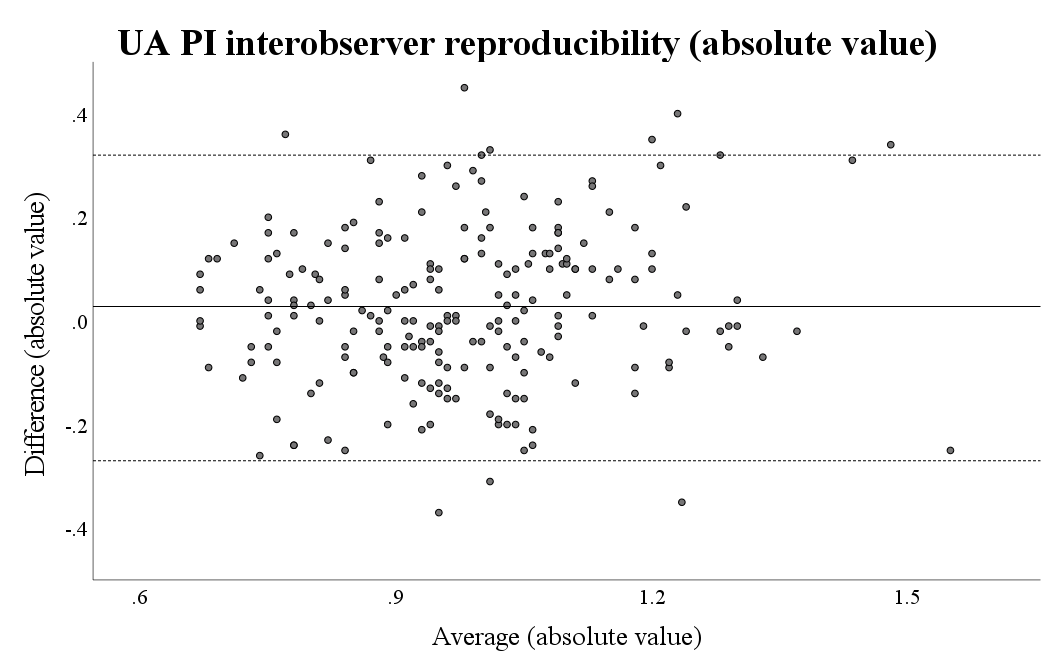

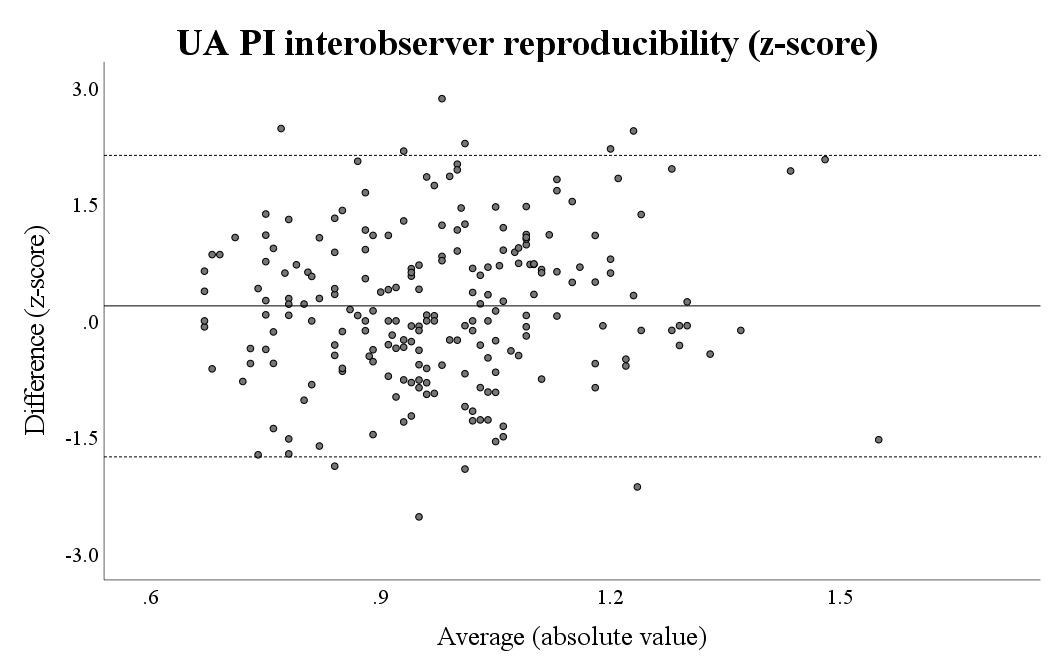

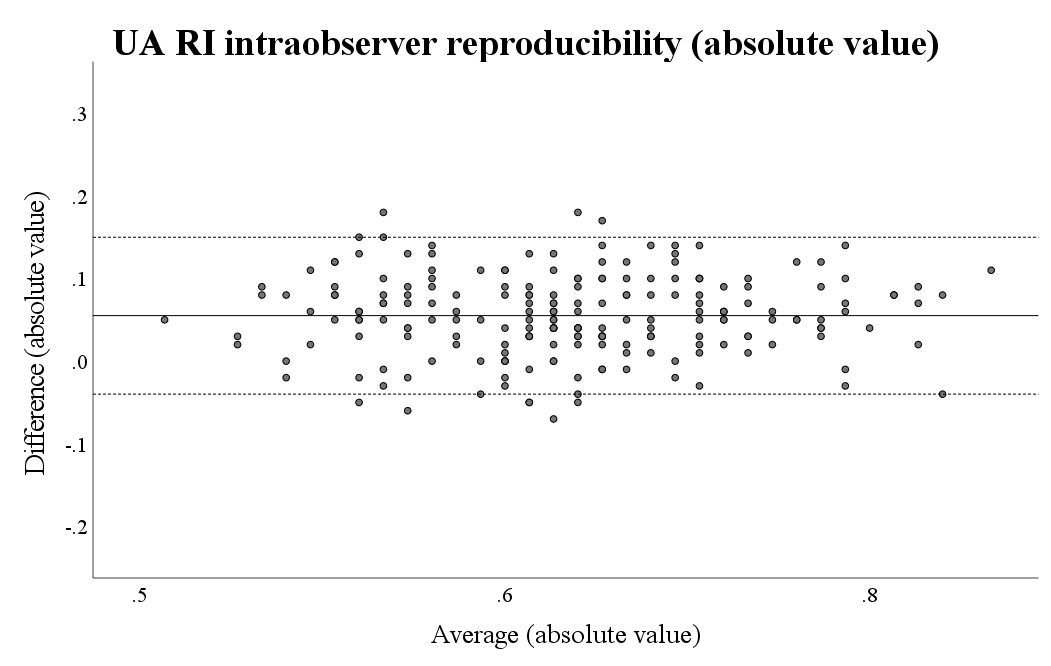

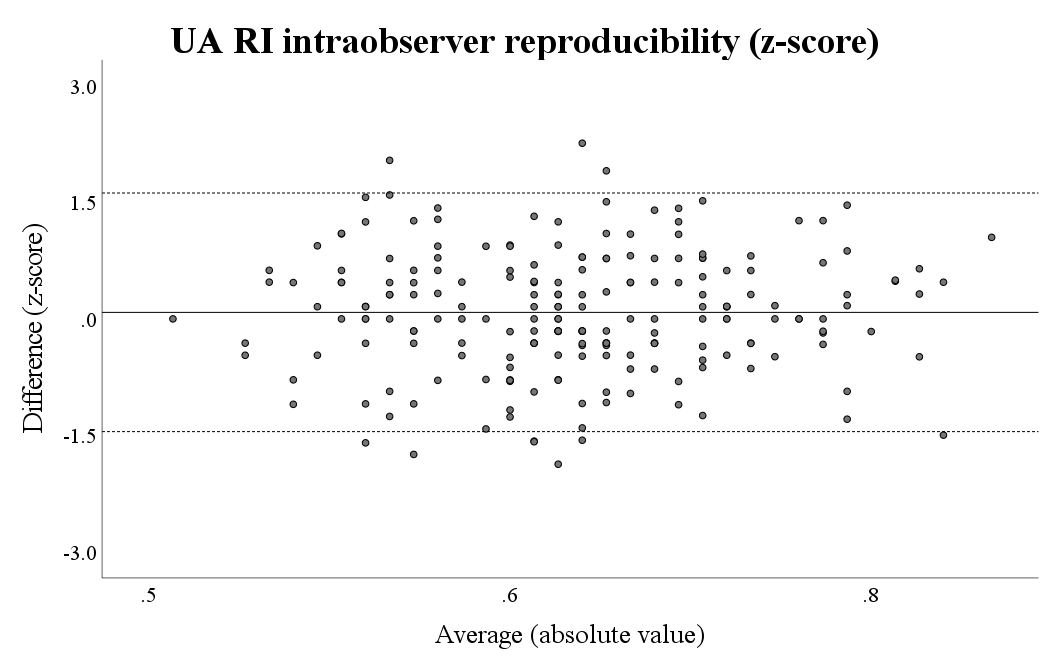

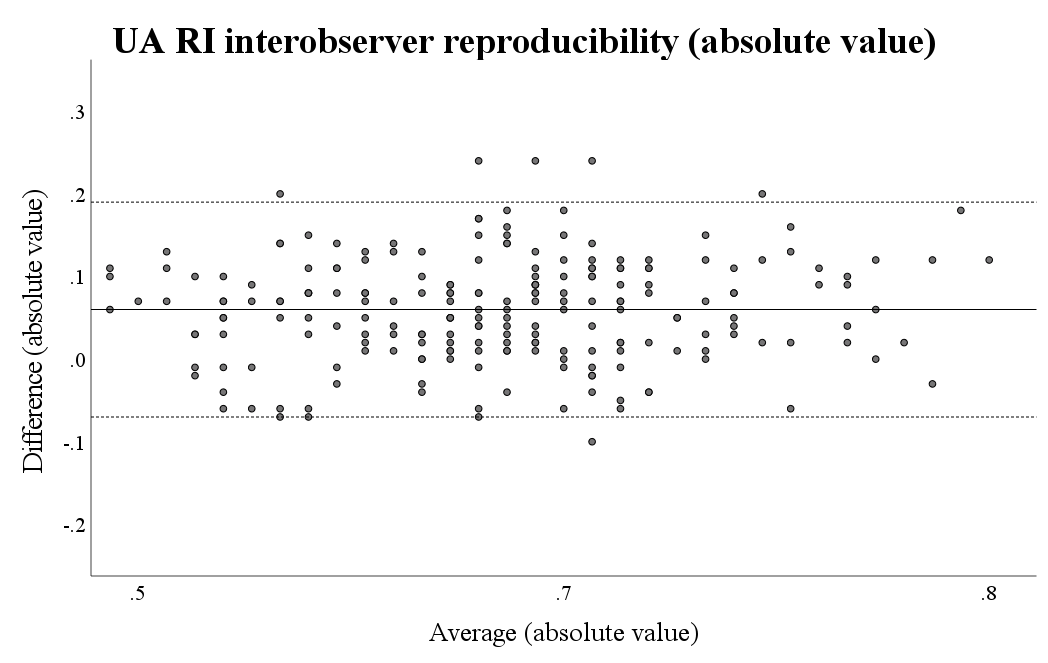

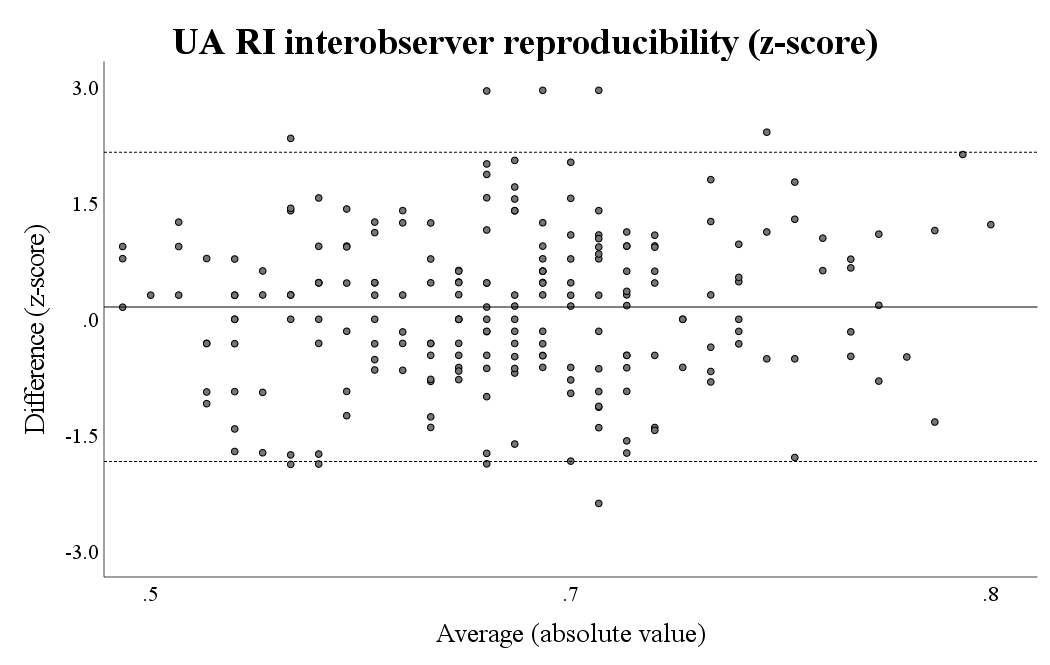


**B) MCA PI, RI and PSV**

**
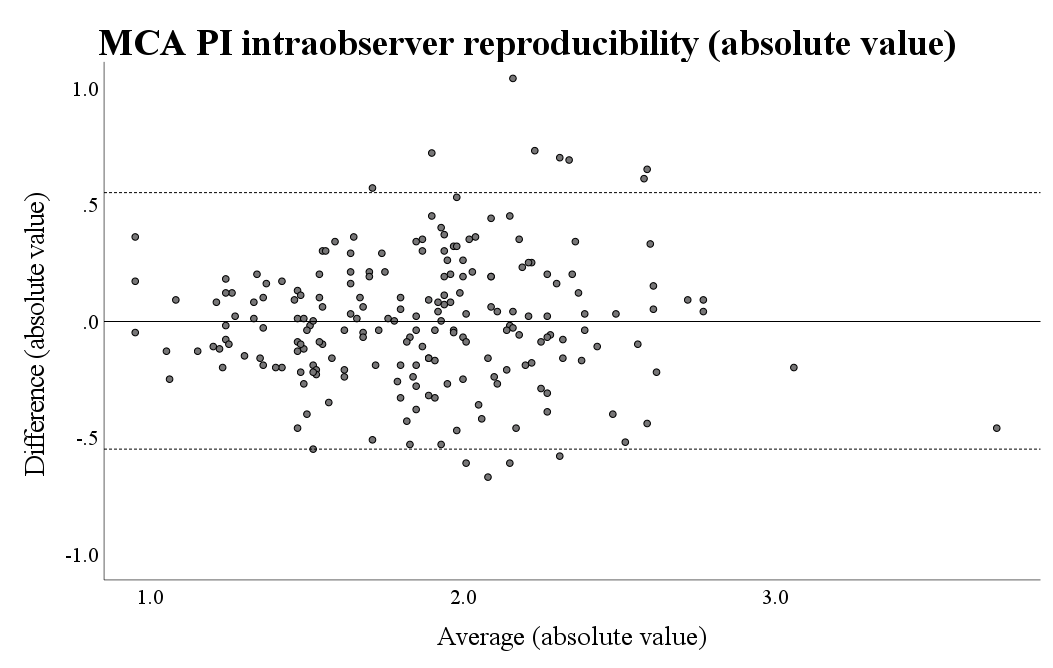
** **
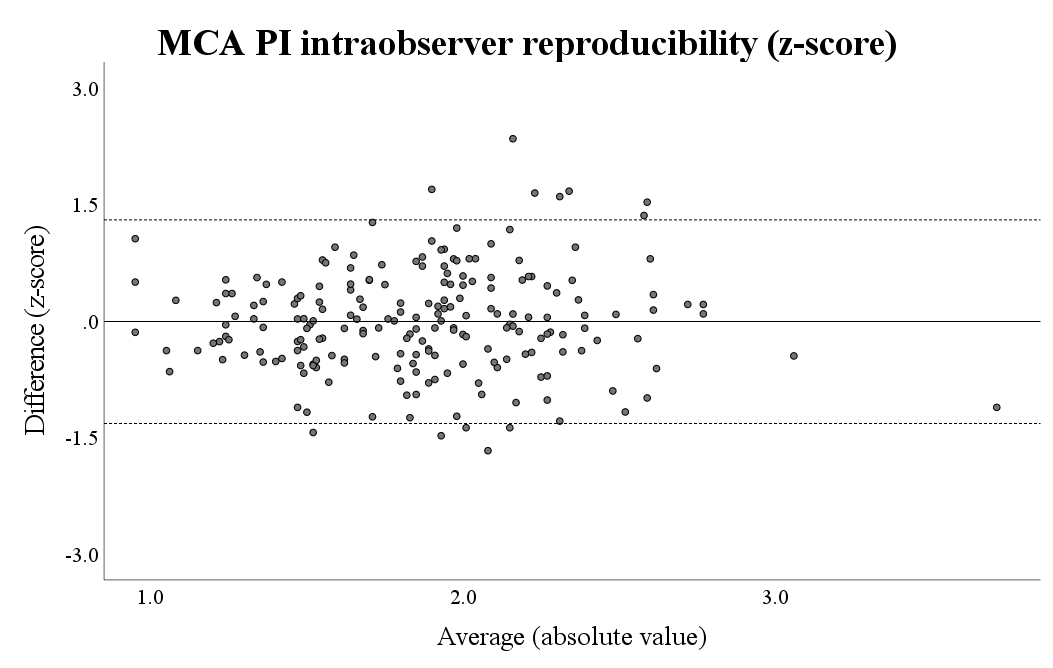
**


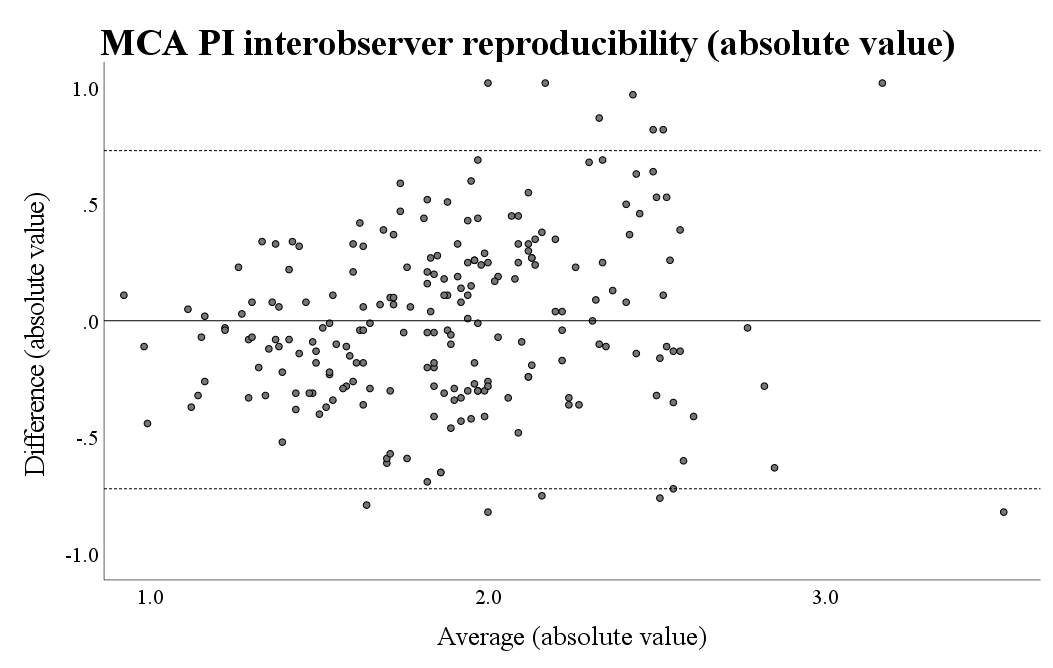

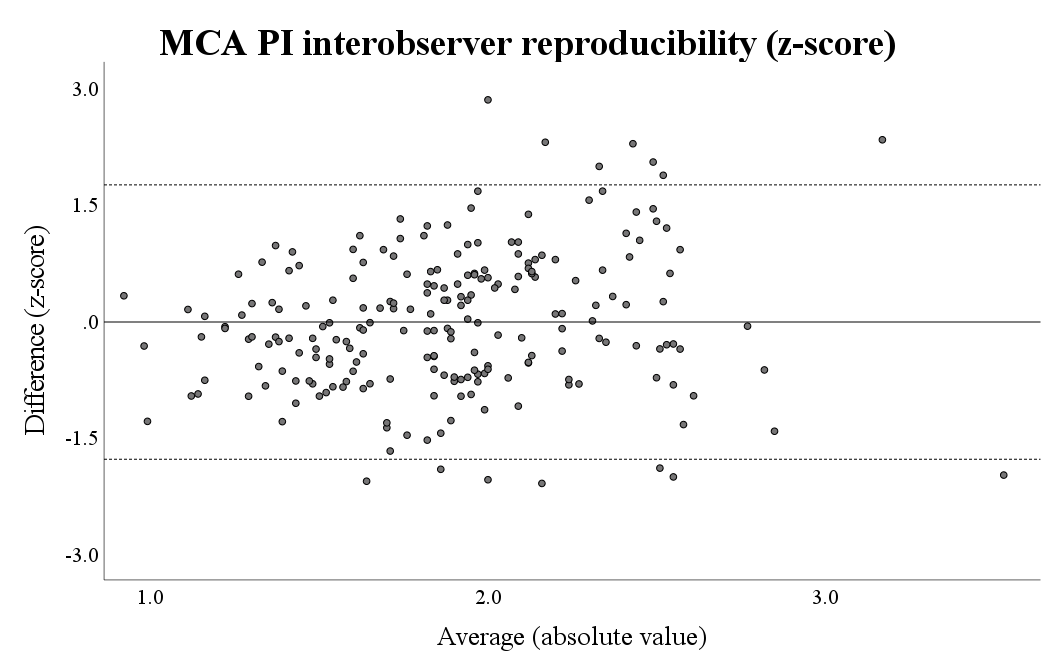


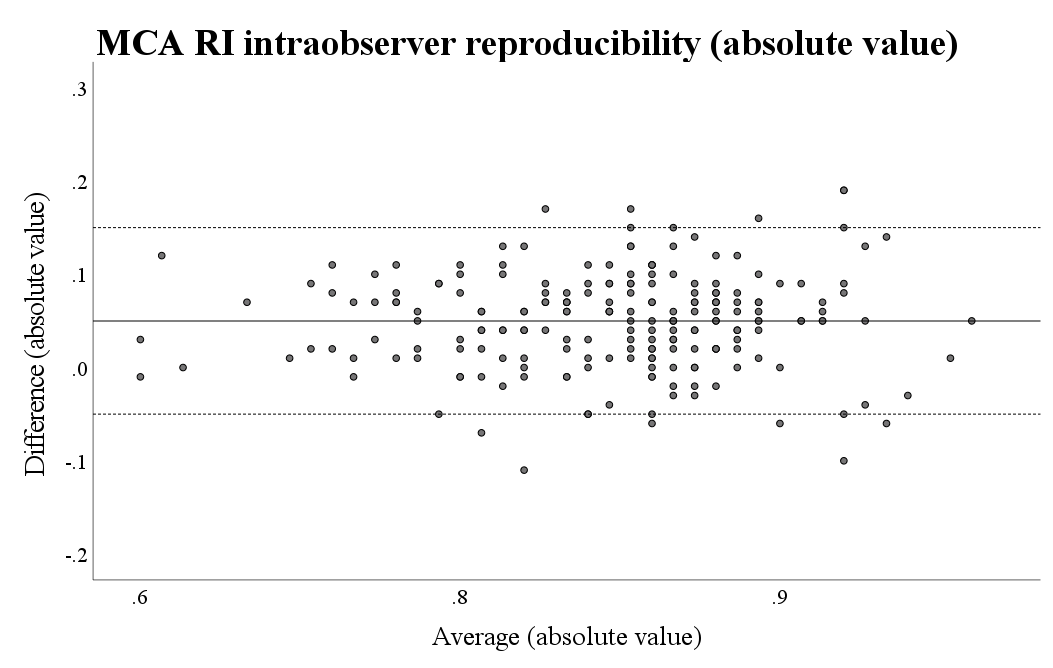


**
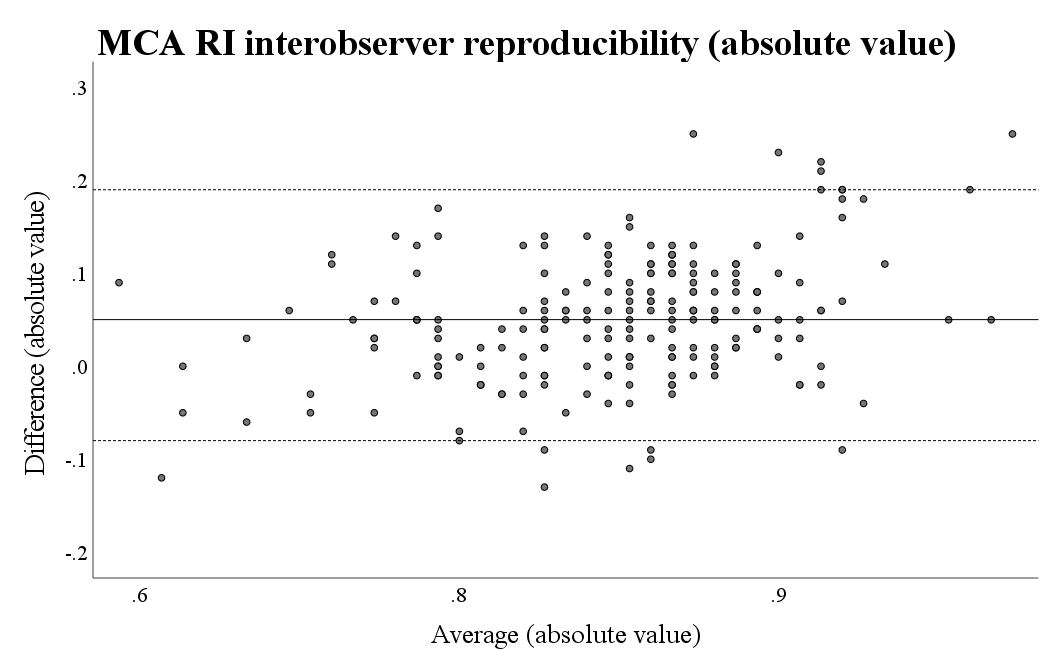
**

**
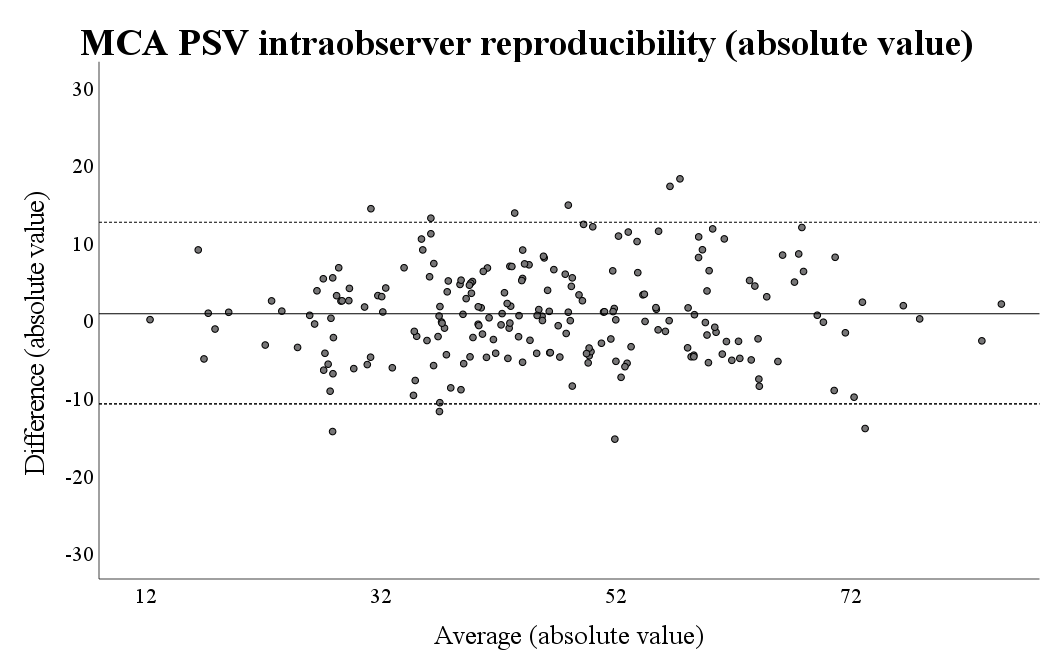
** **
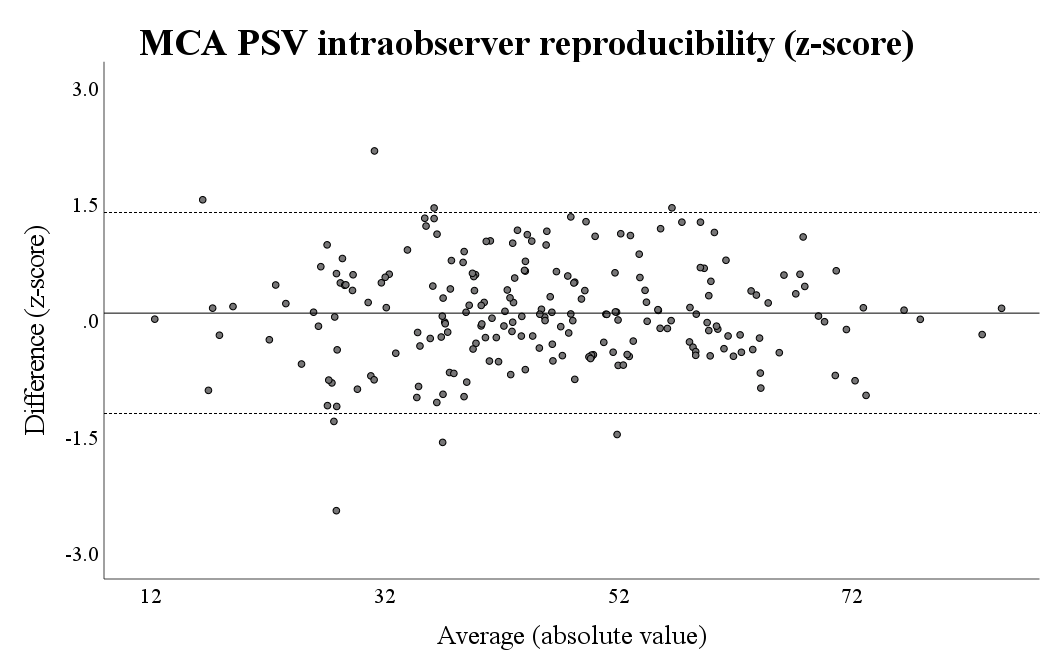
**


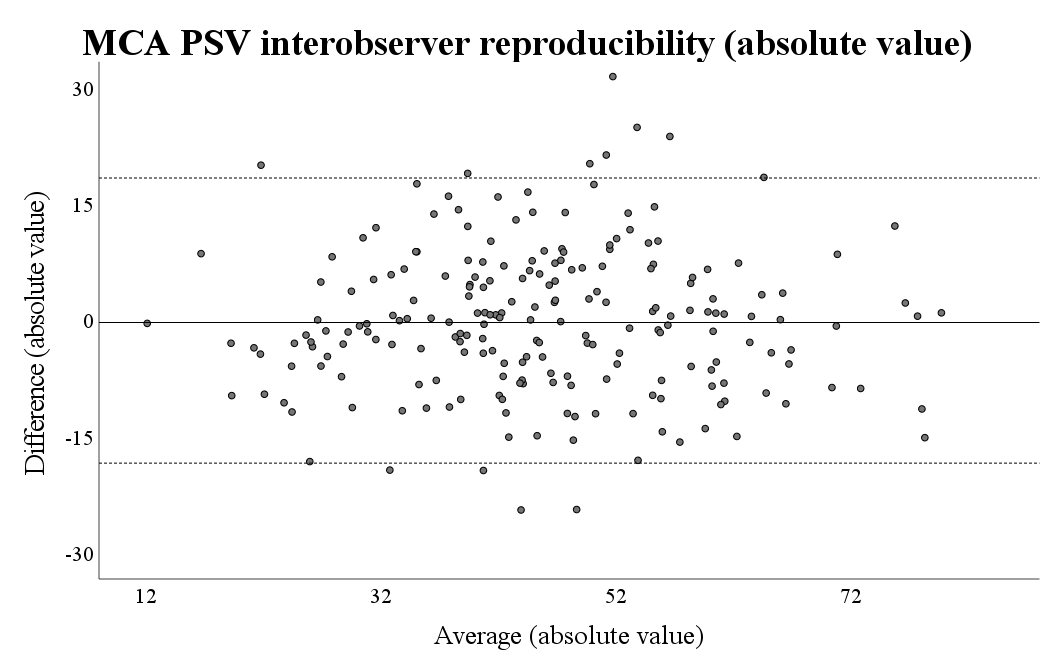

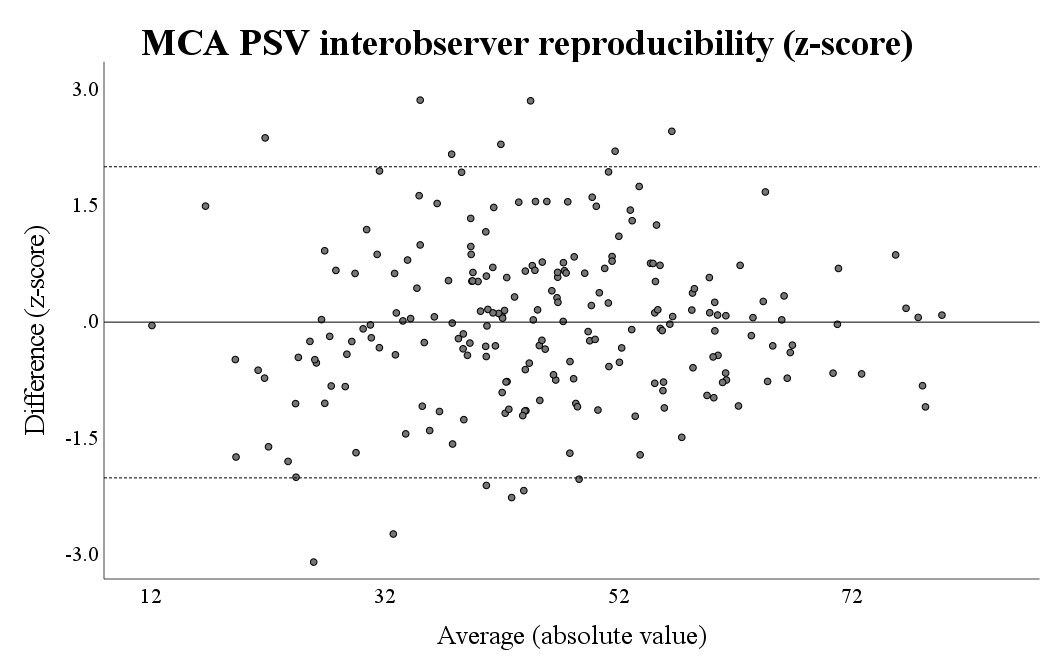


**C) CPR**

**
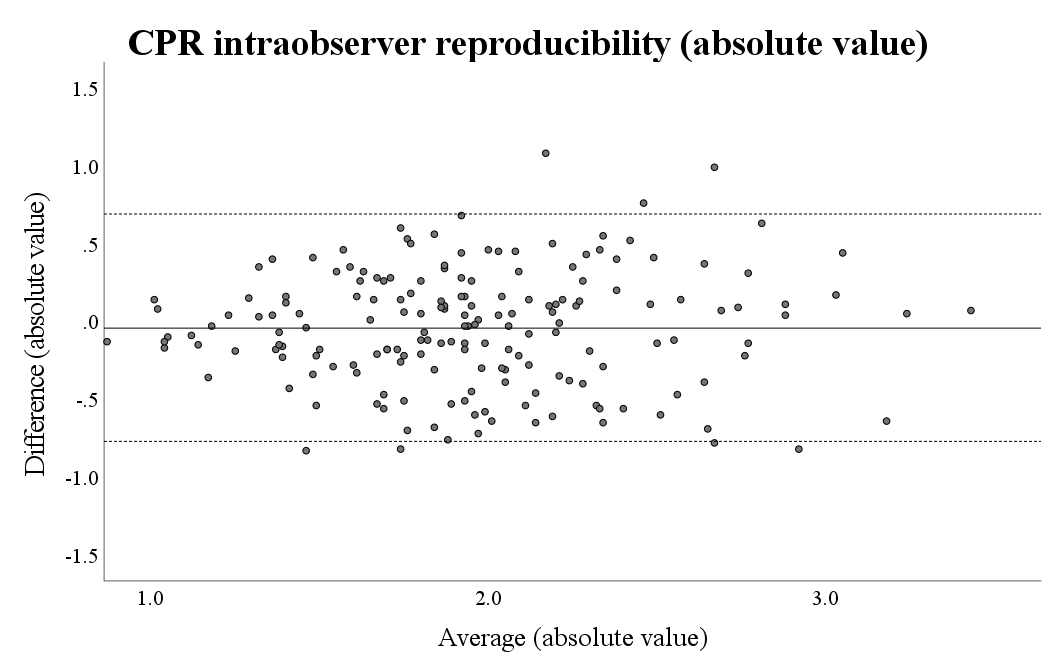
**
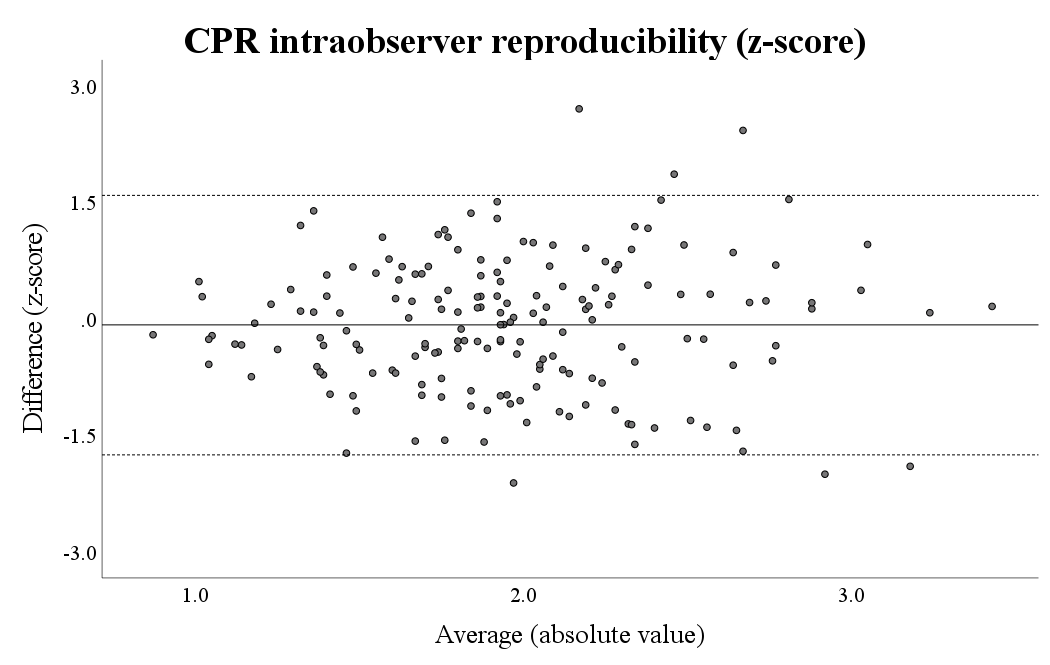


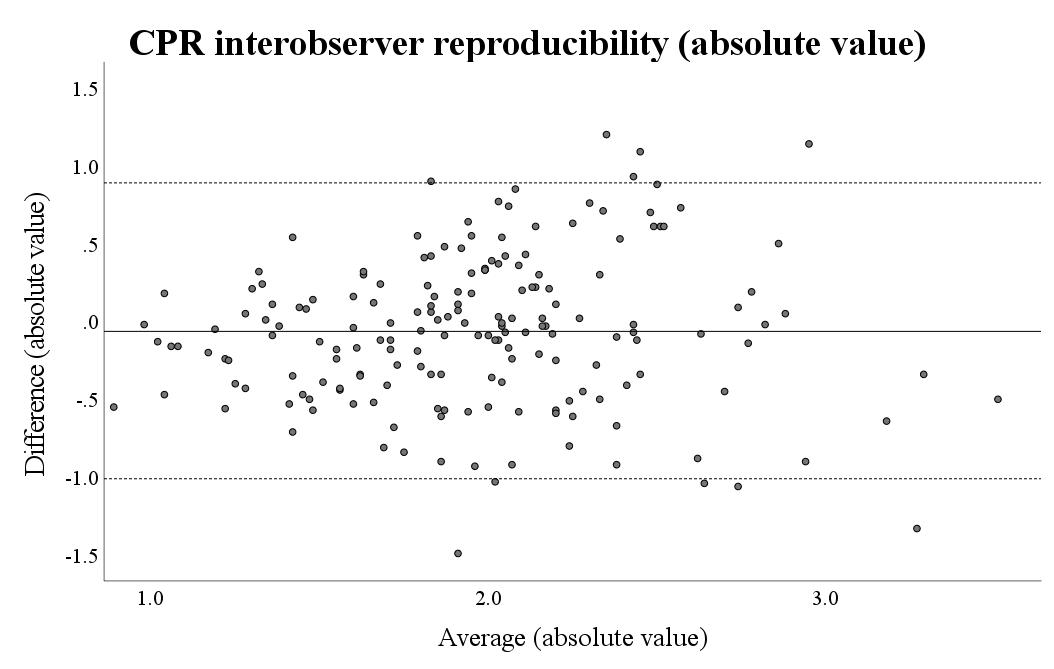

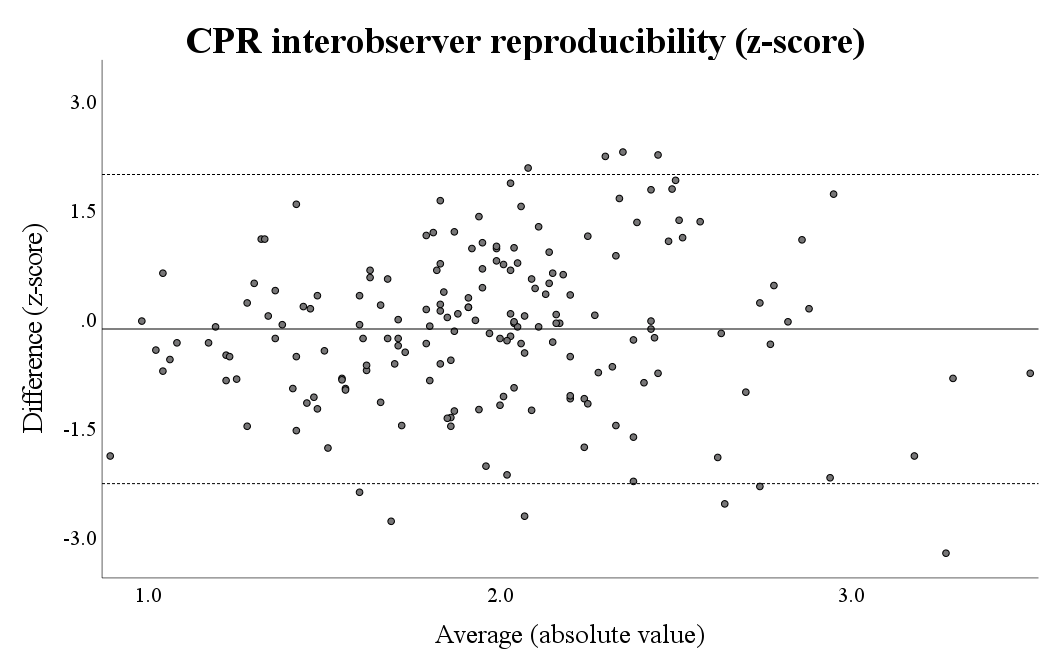


1. **UCR**


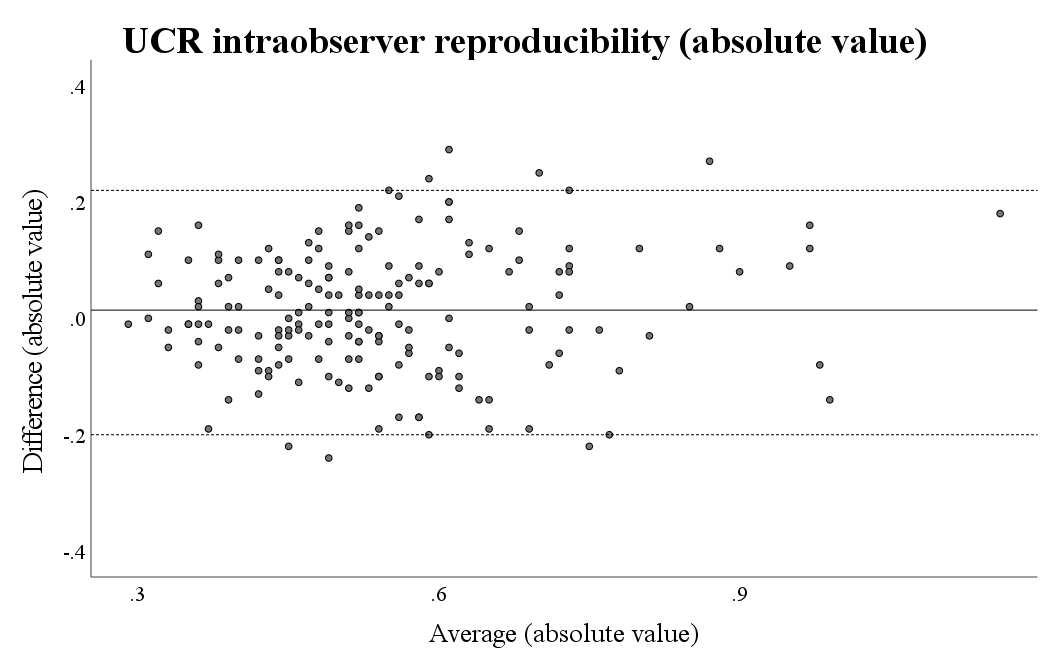

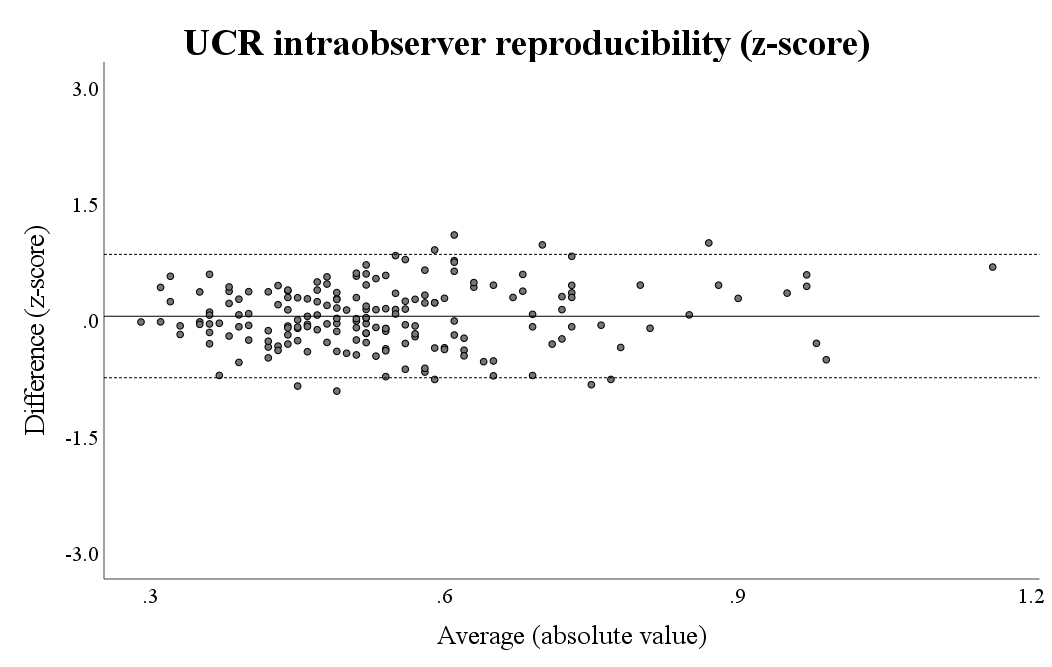


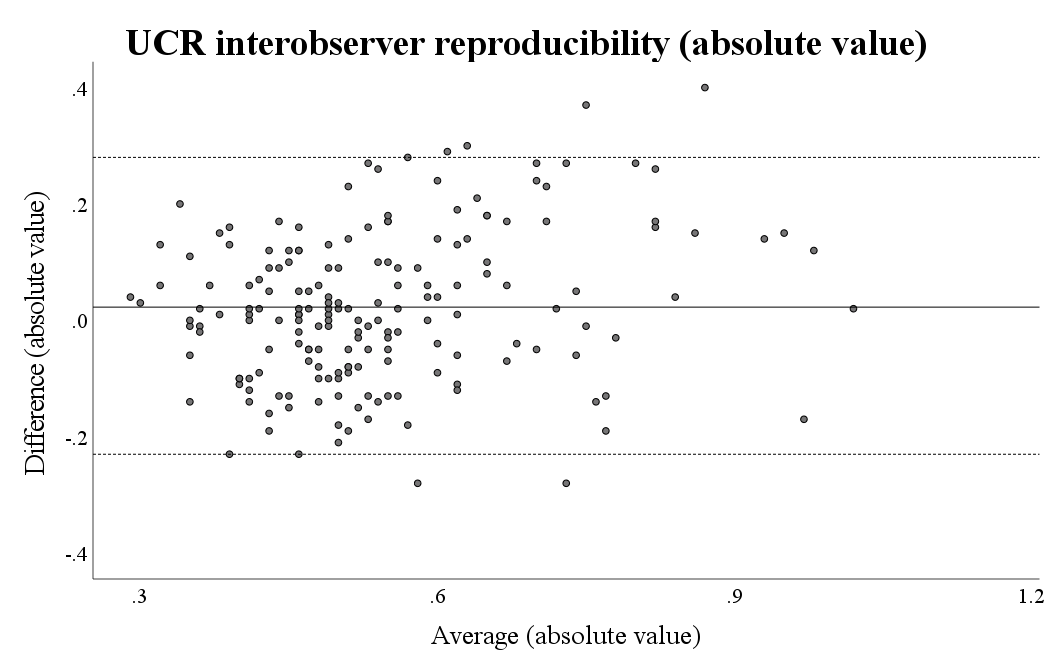

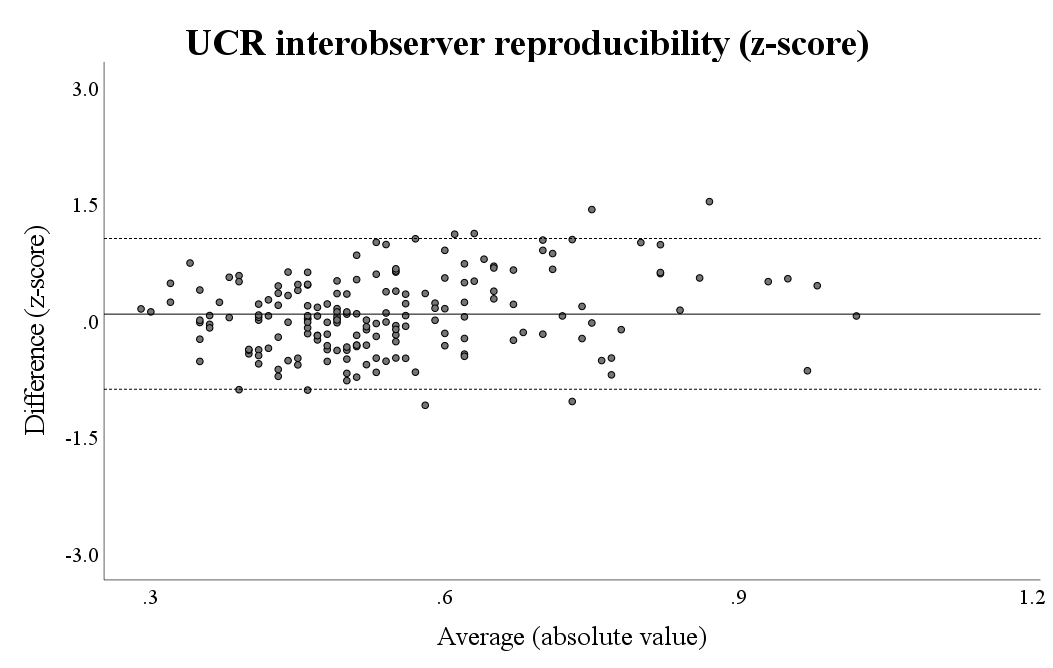

Supplement: Supplementary file 1 — Supplementary file1 (DOCX 1245 KB) [file 404_2024_7883_MOESM1_ESM.docx]
